# The Environmental Costs of Artificial Intelligence for Healthcare

**DOI:** 10.1007/s41649-024-00295-4

**Published:** 2024-06-21

**Authors:** Amelia Katirai

**Affiliations:** Research Center on Ethical, Legal, and Social Issues, Osaka University, Osaka, Japan

**Keywords:** Artificial intelligence, AI, Healthcare, Environment, Planetary Health

## Abstract

Healthcare has emerged as a key setting where expectations are rising for the potential benefits of artificial intelligence (AI), encompassing a range of technologies of varying utility and benefit. This paper argues that, even as the development of AI for healthcare has been pushed forward by a range of public and private actors, insufficient attention has been paid to a key contradiction at the center of AI for healthcare: that its pursuit to improve health is necessarily accompanied by environmental costs which pose risks to human and environmental health—costs which are not necessarily directly borne by those benefiting from the technologies. This perspective paper begins by examining the purported promise of AI in healthcare, contrasting this with the environmental costs which arise across the AI lifecycle, to highlight this contradiction inherent in the pursuit of AI. Its advancement—including in healthcare—is often described through deterministic language that presents it as inevitable. Yet, this paper argues that there is need for recognition of the environmental harm which this pursuit can lead to. Given recent initiatives to incorporate stakeholder involvement into decision-making around AI, the paper closes with a call for an expanded conception of stakeholders in AI for healthcare, to include consideration of those who may be indirectly affected by its development and deployment.

Healthcare is a key setting where expectations are rising for the potentially life-enhancing benefits of artificial intelligence (AI)—“the science and engineering of creating intelligent machines that have the ability to achieve goals like humans via a constellation of technologies” (Topol 2019). Healthcare systems globally have been under strain, exacerbated by the Covid-19 pandemic, and worsened by high levels of burden, issues of work-life balance, and an aging workforce (Topol 2019; Obermeyer and Lee 2017; Meskó et al. 2018). At the same time, with the rise of big data, “the complexity of medicine now exceeds the human mind,” as healthcare professionals struggle to make time for the “deep thinking” (Obermeyer and Lee 2017) that is required of them, squeezed as they are between competing pressures. Meanwhile, healthcare services in many global settings remain inadequate, with individuals lacking access to safe and affordable essential care (Meskó et al. 2018).

Under these strained circumstances, there is a strong tendency towards looking to emerging technologies as a solution (Morozov 2013; Sætra 2023), with AI appearing to be almost a panacea (Davis 2020). There is a growing perception of AI as the best hope for harnessing the complexity of medicine and ensuring a higher quality of healthcare, with predictions that AI will become “indispensable” (Obermeyer and Emanuel 2016). Yet, there is a contradiction at the center of AI for healthcare: that its promise lies in its expected improvements to its human health, while its widespread development and implementation will carry heavy environmental costs, to the detriment of the health of many of the most vulnerable on the planet (King and Harrington 2018)—often those who will be left bereft of its possible benefits. This contradiction is not within the health area alone—as noted by Nordgren (Nordgren 2023), there is a “dual role of AI in relation to climate change” in that it contributes to climate change, while also potentially helping to mitigate it (Owe and Baum 2021a; Coeckelbergh 2021). This contradiction has direct implications for human health and well-being, yet has remained largely obscured in relation to healthcare (Coghlan and Quinn 2023). This has occurred at both a conceptual level and a practical one, as reflected in, for example, a recent scoping review on literature related to ethics on AI in health which did not address the health costs arising from the environmental impact of AI despite its focus on the need to “mitigate … potential harms, especially for the most vulnerable” (Murphy et al. 2021; see also Char et al. 2018; Gerke et al. 2020; Martinez-Martin et al. 2021; Hadley et al. 2020), or to offer a second example, in an examination of one set of national plans for AI in healthcare, which found no reference to environmental considerations (Katirai 2023).

In this paper, this contradiction—between the purported benefits of AI for healthcare and the environmental costs which ultimately harm health—is critically interrogated. It is noteworthy that “AI” is used here to refer to, as in the definition above, a “constellation” of technologies, of varying clinical utility and social benefit. The term AI itself has come under critique for its ambiguity and “nebulous” (Katz 2020) nature (Crawford 2021), and there are inherent limitations to discussing the implications of a collection of technologies through this singular framing, given that there is variability among the technologies referred to. However, this overarching category, often used in outward-facing promotions of the technologies (Crawford 2021), is used here to collectively represent these technologies, and to underscore commonalities in the environmental burdens they pose.

## The Expected Benefits of AI for Healthcare

There is hope that the implementation of AI will lead to more equitable access to, and a higher quality of, healthcare within and across countries, and particularly in low-resource settings. To offer just a few examples, this includes expectations that AI-based expert systems can provide guidance to clinicians where a specialist is inaccessible; that AI can help in appropriately allocating existing healthcare resources; and that it can help to prepare for outbreaks of infectious diseases (Wahl et al. 2018). Where healthcare is already available at a relatively high level, there are hopes that AI will further raise the standard of both preventive care and treatment, through advanced screening techniques, increased accuracy in pathology and radiology, and the promotion of personalized medicine (Topol 2019; Danku et al. 2022). Through this, a key hope is that the burden on healthcare professionals will be reduced, creating space for the more intrinsically “human” work of caring for patients (Topol 2019), with AI even serving as a “tool to truly understand patients” (Obermeyer and Emanuel 2016).

As Topol (2019) writes:

The promise of artificial intelligence in medicine is to provide composite, panoramic views of individuals’ medical data; to improve decision making; to avoid errors such as misdiagnosis and unnecessary procedures; to help in the ordering and interpretation of appropriate tests; and to recommend treatment. Underlying all of this is data.

Indeed, data is critical to the development of AI for healthcare, as machine learning algorithms are “data hungry,” requiring “millions of observations” (Obermeyer and Emanuel 2016) in order to provide output—observations which are increasingly available not only through clinical records, but also through a range of wearable devices (Schüll 2016). Though healthcare data is often described as an objective reflection of an individual’s state of health, documented biases and diagnostic errors remain rampant, calling into question the bases of these technologies (Topol 2019; Joyce et al. 2021).

There is a rapidly expanding industry around the promise of AI, which is receiving major investment from both the public and private sectors, particularly in highly industrialized nations (e.g., Katirai 2023). AI is often framed in discourses of “social good,” without sufficient recognition that what is “good” may be defined differently depending on one’s position in society (Madianou 2021), and that even AI developed for such “good” purposes may simultaneously have harmful outcomes (Astobiza et al. 2021).

van Wynsberghe (2021) has conceptualized AI ethics as occurring in three waves. Though there has arguably been persistence in these themes across and beyond their distinctive waves, the conceptualization points to key—though non-exhaustive—areas of concern regarding AI. These include the possible loss of control of AI and its development to the point of eventual superiority over humans, and issues of bias and inequality, which are believed to be (to some debatable extent) remediable through technological fixes and improved data sets (Joyce et al. 2021; Gebru 2020). However, societal and academic concern over these issues has in some measure sidestepped questions about the justifiability of the pursuit of AI, and have glossed over critiques of technological determinism and the projected inevitability of AI advancement (Benjamin 2019; Zuboff 2019; Hagendorff 2022). As Hagendorff (2022) has argued, “AI ethics should definitively dare to become more ‘radical.’” These critiques have come into sharper focus through arguments such as van Wynsberghe’s, which redirect questions about how AI can contribute to more sustainable societies (e.g., Cabinet Secretariat of Japan 2019) towards a reconsideration of the sustainability and environmental costs of AI itself (Crawford 2021; van Wynsberghe 2021; Brevini 2021; Brevini 2023; Brevini 2020; Robbins and van Wynsberghe 2022).

There has been a recent emphasis on “human-centered AI,” with the use of AI to meet sustainability goals embedded in principles for its design and implementation (Cabinet Secretariat of Japan 2019; Rigley et al. 2023). As Pasquale (2020) has argued, it is undoubtedly important to ensure that human expertise is valued, and that AI is designed to benefit and not harm humans. Yet this perspective also reflects anthropocentrism (Rigley et al. 2023; Coghlan and Parker 2023; Bossert and Hagendorff 2023; Hagendorff et al. 2023) as well as human supremacy (Estrada 2020) and an orientation away from ecological justice (Donhauser 2019), and risks obscuring the potential harm of AI for healthcare to the global environment and to nonhumans. As documented by Owe and Baum (2021b; 2023), anthropocentrism may take multiple forms. It may include ontological anthropocentrism or “human/nature dualism,” the embrace of which leads people to “damage nature in ways that ultimately hurt themselves”—as will be seen in the case of AI in healthcare discussed below (Owe and Baum 2021b). It may also take the form of ethical anthropocentrism—“the idea that humans are more intrinsically valuable because they are humans,” which may manifest in a weak or moderate (Rigley et al. 2023) form which recognizes “some intrinsic value of nonhumans,” but in its strong form “rejects moral consideration of nonhumans” (Owe and Baum 2021b) entirely.^1^ As Owe and Baum (2021b) argue, each of these forms of anthropocentrism should be rejected, and should not be perpetuated through mere thoughtlessness (Baum and Owe 2023), particularly as disregarding the interests of nonhumans is “ethically wrong” (Hagendorff 2022).

This re-evaluation is essential in the face of mounting evidence that these forms of anthropocentrism have not only devastated the global environment and led to the irretrievable loss of biodiversity and the vast array of individual lives this represents, but ultimately have come to damage our own well-being (Owe and Baum 2021b; Bossert and Hagendorff 2023; Marselle et al. 2019; Ross 2020).^2^ This error should be avoided with regard to AI in healthcare, the very aim of which is ostensibly to benefit human health. As Brevini (2021; Baum and Owe 2023) has pithily expressed it: “If we lose our environment, we lose our planet and our lives. So we must understand and debate the environmental costs of AI.” As argued above, to date, there has been insufficient consideration of these costs in relation to the AI that is used for healthcare, and it is thus the focus below.

## Environmental Costs Across the Lifecycle

The environmental costs of the development of AI extend across the lifecycle (Crawford 2021; Brevini 2021; Taffel et al. 2022), yet, despite the pursuit of AI for healthcare, there has been a notable lack of attention to these costs in this context (Coghlan and Quinn 2023). As Crawford (2021; Crawford and Joler 2018) documents, the costs of AI begin from the pollution and degradation of the environment resulting from the creation of the hardware to power AI. For example, rare earth metals are critical to a range of hardware required for AI including capacitors, fuel cells, and insulation, and can be found in devices and batteries powering AI; they are also critical to processes of miniaturization which make it possible for AI processing to be portable (Crawford 2021; Taffel et al. 2022; Pitron 2020). These metals are often extracted in ways which are devastating both to humans and to the environment, in situations involving conflict, major human rights abuses, and the loss of life (Crawford 2021; Kara 2023). Processing a single ton of rare earth ores can produce up to two thousand tons of toxic waste, which is left to pollute the natural environment (Crawford 2021). Indeed, rare earth metals are not only “rare” because they are hard to find, but because they are so difficult and dangerous to extract (Crawford 2021; Butters 2016). Despite this, estimates suggest that just 1% of the metals are recycled, meaning that the majority of required metals must be newly extracted from the earth (Crawford 2021). This is particularly problematic given that the strong demand for such metals is being outstripped by a dwindling accessible supply (Crawford 2021; Pitron 2020).

Moving on to other parts of the lifecycle, data centers used to power AI guzzle electricity and water to keep them cool and functioning (Crawford 2021; Brevini 2021; Stokel-Walker 2022; Dauvergne 2020). These processes are becoming increasingly problematic as global temperatures rise and heat waves strike, making them both a threat through, and under threat from, climate change (Stokel-Walker 2022). Yet, estimates suggest that:

by 2025, the ICT industry could consume 20 percent of the world’s electricity (up from 3 to 5 percent in 2015) and account for more than 5 percent of global carbon emissions. By then, without a sizable increase in energy efficiency and renewable electricity, data centers alone could account for over 3 percent of global carbon emissions (four-fifths of data centers were using fossil-fuel electricity in 2018). (Hagendorff et al. 2023; Dauvergne 2020)

There are hopes that some of this may be offset by a shift to “clean” energy—though increased growth in the uses of these technologies often lead to increased energy demand, posing challenges to a full and rapid transition to renewable energy (Hickel 2021). Moreover, as highlighted by Pitron (2020), these energy sources themselves rely on rare earth metals, and carry their own environmental costs. Nonetheless, companies have been working to increase energy efficiency and decrease energy consumption, claiming to move beyond carbon neutrality, as in the case of Google, for example, which reached carbon neutrality in 2007 (Dauvergne 2020). Yet, data suggests that Google’s data centers still draw electricity from power plants reliant on fossil fuels, and Google has argued that powering data centers with entirely renewable energy remains impossible at present; they offset this footprint by purchasing renewable power credits (Dauvergne 2020). This is even as concerns are rising that major carbon offset schemes may not only have little positive impact, but may actively be harmful to the environment (Lakhani 2023).

Then, there is a need to consider the massive amounts of carbon dioxide that are emitted through the development and fine-tuning of algorithms (Cowls et al. 2023). Strubell et al. (2019) found that training for a single deep learning-based, natural language processing algorithm led to over 270,000 kg of carbon emissions. This does not include the emissions that would be required for any subsequent training of an algorithm, for example to increase its inclusivity (van Wynsberghe 2021).^3^

Yet, returning to perspectives from the initial stages of the lifecycle, the physical infrastructure powering AI will extend beyond that of the “immaterial” (Jaume-Palasi 2019) algorithms themselves. Ultimately, as Crawford (2021) has tersely expressed it:

Each object in the extended network of an AI system, from network routers to batteries to data centers, is built using elements that required billions of years to form inside the earth. From the perspective of deep time, we are extracting Earth’s geological history to serve a split second of contemporary technological time, building devices … that are often designed to last for only a few years.

What becomes of devices which have reached the end of limited lifespans, through planned obsolescence and in the pursuit of more powerful algorithms and devices to run them (Dauvergne 2020)? Estimates suggest that just one-fifth of the world’s “e-waste” is safely recycled (Dauvergne 2020), and that despite efforts to legislate e-waste, the rest is dumped in resource-poor settings, where human beings attempt to recover parts of value, exposed to toxic chemicals which leach into the environment as the devices are buried in landfills or burned in pits; ultimately, “the e-waste produced by AI-enabled devices all eventually profoundly damage animal habitats” (Coghlan and Parker 2023; Parvez et al. 2021). The amount of global e-waste grew from 35 million metric tons in 2010, to 50 million metric tons in 2018, and is estimated to reach as much as 120 million metric tons by 2050 (Dauvergne 2020).

## A Critical Contradiction

This creates an inherent contradiction. A range of technologies under the umbrella of AI are being developed for healthcare, with high expectations for the ways in which they can bring improvements to human health, though it remains unclear to what extent this pursuit will bring significant and justifiable improvements over the status quo in practice, even as commercially driven development continues apace (Ishii et al. 2020; Marwaha and Kvedar 2022). Despite this, the development and implementation of AI as it occurs today with insufficient oversight and safeguards for environmental costs creates new health vulnerabilities across its lifecycle–vulnerabilities which are concentrated on the already vulnerable (Coeckelbergh 2021), whether human or nonhuman. As Marselle et al. (2019) have written in relation to human vulnerability:

Climate change poses significant challenges to human health and biodiversity. Increased numbers of heat waves, droughts and flooding events due to climate change have negative consequences for both human health and biodiversity. … The most vulnerable people in society – the elderly, those with chronic diseases and persons of lower socio-economic status – are often most affected.

These impacts include, for example, findings reported in a review from the *Lancet* Commission on pollution and health (Fuller et al. 2022) that pollution continued to be responsible for 9 million deaths per year—one in six deaths worldwide. While the number of deaths from pollution in the household and pollution in water have been in decline, there have been rises in deaths due to air pollution and toxic chemical pollution—a 7% rise since 2015, and a 66% rise since 2000. Though AI is clearly not the primary entity responsible for this pollution, commitments to carbon neutrality by developers of AI as described above obscure these ongoing planetary and pollution-related costs, which lead to an increased incidence both of mortality and of lowered quality of life.

Meanwhile, estimates suggest that despite global accords such as the Paris Agreement, the world is on target to experience significant heating by the end of the century, with predictions in even “the most optimistic scenario” (UN Environment Programme 2023) indicating an increase in heating of between 1.8 and 2.5 °C. Yet, even an increase of a single °C in global temperature has been found to lead to excess mortality of up to 12% (Marselle et al. 2019). These seemingly abstract figures and the feedback loops they trigger also have immediate, individual costs, including, for example, those whose lives are directly impacted by agricultural losses (Wallace-Wells 2019; Carleton 2017). These risks may also be largely unnoticed, such as through the impact on health and well-being of a loss of “common types of nature experience” which “are decreasing in quantity and quality for many people around the globe” (Bratman et al. 2019).

By allowing the development of AI for healthcare to be commercially driven and to be pursued based on what is possible rather than what is needed and beneficial (Hicks 2021), we risk becoming locked into a vicious cycle through which degradation of the natural environment leads to further harm to human health, through the very attempts to optimize it. It is noteworthy that the harm to human health which occurs is often at a remove from those who are benefitted by the technology (Mulligan and Elaluf-Calderwood 2022), as can be seen through the example of those exposed to the toxic aftermath of e-waste described above.

Zuboff (2019) describes the way in which “inevitability” takes hold and creates a perception of technological development as an onrushing river which we cannot or should not stem (Sætra 2023). This is true in the case of AI for medical purposes as well. Healthcare is an application for AI for which the case for AI development is strong, particularly if certain applications can be proven to bring a tangible, and ultimately quantifiable, benefit to the lives of individuals. It is an arena for the application of AI which is seen to be for the greater good rather than for the benefit of particular companies—a perspective which, however, serves to obscure the role of commercial interests behind its pursuit (Katirai 2023). Yet, discussions of the utility of AI are increasingly held in a vacuum, where they are disassociated from its costs, and from the extractive logics behind the technologies; this means that these discussions often occur without consideration of proportionality and whether the technologies indeed merit their environmental and social costs (Karliuk 2022).

This is significant because the development of AI, particularly when it is promoted with public funds (e.g., 13), can operate as a zero-sum game, wherein prioritizing the development of AI means that reduced funds are available in other ways. As Pasquale (2020) argues, we can envision other possible futures and other possible avenues for investment which lead to benefits in human health. There is, of course, much beyond AI and emerging technologies which is harmful to the environment and natural ecosystems. Yet, before we massively invest in, and reshape our societies around, emerging technologies, there is a duty to reflect on the possible long-term implications of these technologies, including their environmental impact.

In light of a recent push towards increased recognition of the stakeholders in healthcare (WHO 2021; Banerjee and Griffiths 2023; Banerjee et al. 2022), I argue that this must also involve a shift in who we understand these stakeholders to be. Our understanding of stakeholders must extend beyond those who are directly impacted by AI development as it comes to be incorporated in clinical workflows and in daily lives, to consider those who may never be given the opportunity to be involved in these dialogues. What will the impact be on those who are indirectly affected by it? On those who will handle the e-waste that will inevitably arise from these pursuits? And those who will suffer in a degraded climate as carbon emissions continue to grow?

We must take this line of reasoning further. Our anthropocentric, “human-centric” (Cabinet Secretariat of Japan 2019) visions of AI exclude not only the stakeholders who are not (Mulligan and Elaluf-Calderwood 2022; Benjamin 2013), but also those who by definition *cannot* be, at the table (Rigley et al. 2023). We must consider the impact of the pursuit of these technologies on biodiversity, even as we have lost two-thirds of the abundance of species over the last half-century, without fully understanding the feedback loops that may be triggered by its loss (World Economic Forum 2022; Román-Palacios and Wiens 2020; Donhauser et al. 2021) and their implications for future generations (Halsband 2022). We must move a step beyond socio-technical, human-oriented visions of AI—looking beyond how technologies are embedded in society, to see how they are embedded in even broader ecosystems, with those whose stake in these matters is their very lives. We must reconsider how the visions which we prioritize and pursue for the improvement of our own lives may threaten those of a myriad others—others who we will ultimately find, perhaps once they are irretrievably lost, are indispensable for our own well-being (Coghlan and Parker 2023). It is essential that we move beyond the technologies themselves and take time to consider their broader societal and environmental implications (Benjamin 2019). What is needed are clear-headed evaluations of the promise and pitfalls of emerging technologies, through processes that involve a diverse range of voices. This will require, in an urgently needed next step, a reimagining of evaluations for the use of AI, and a widening of the scope of concern for its implications.

## Footnotes

1 To this, Scheessele (2022) and others add conceptual anthropocentrism, denoting the existence of a uniquely human view of the world.

2 Though, as noted by Owe and Baum (2021b) in the context of sustainability and AI, “it is not enough to sustain nonhumans for their instrumental role for humans”.

3 Further, there may be harm which results from particular applications of the technologies—as discussed by Coghlan and Parker (2023), for example, the use of AI to identify new treatments or vaccinations would then lead to animal testing, which may lead to harm.
